# Kinetics of 2 different high-sensitive troponins during targeted temperature management in out-of-hospital cardiac arrest patients with acute myocardial infarction: a post hoc sub-study of a randomised clinical trial

**DOI:** 10.1186/s12872-022-02778-4

**Published:** 2022-07-30

**Authors:** Alf Inge Larsen, Anders Morten Grejs, Simon Tilma Vistisen, Kristian Strand, Øyvind Skadberg, Anni Nørgaard Jeppesen, Christophe H. V. Duez, Hans Kirkegaard, Eldar Søreide

**Affiliations:** 1 Department of Cardiology, Stavanger University Hospital, Stavanger, Norway; 2 Department of Clinical Sciences, University of Bergen, Bergen, Norway; 3 Department of Clinical Medicine, Aarhus University, Aarhus, Denmark; 4 Department of Intensive Care Medicine, Aarhus University Hospital, Aarhus, Denmark; 5 Critical Care and Anaesthesiology Research Group, Stavanger University Hospital, Stavanger, Norway; 6 Laboratory of Clinical Biochemistry, Stavanger University Hospital, Stavanger, Norway; 7 Research Centre for Emergency Medicine, Emergency Department, Aarhus University Hospital, Aarhus, Denmark; 8 Division for Heart- Lung- and Vascular Surgery, Anaesthesiology section, Aarhus University Hospital, Aarhus, Denmark; 9 Department of Intensive Care, Stavanger University Hospital, Stavanger, Norway

**Keywords:** Out of hospital cardiac arrest, Myocardial Infarction, Troponins, Targeted temperature management

## Abstract

**Introduction:**

Short term hypothermia has been suggested to have cardio protective properties in acute myocardial infarction (AMI) by reducing infarct size as assessed by troponins. There are limited data on the kinetics of these biomarkers in comatose out-of-hospital cardiac arrest (OHCA) patients, with and without AMI, undergoing targeted temperature management (TTM) in the ICU.

**Purpose:**

The aim of this post hoc analyses was to evaluate and compare the kinetics of two high-sensitivity cardiac troponins in OHCA survivors, with and without acute myocardial infarction (AMI), during TTM of different durations [24 h (standard) vs. 48 h (prolonged)].

**Methods:**

In a sub-cohort (n = 114) of the international, multicentre, randomized controlled study “TTH48” we measured high-sensitive troponin T **(**hs-cTnT), high-sensitive troponin I **(**hs-cTnI) and CK-MB at the following time points: Arrival, 24 h, 48 h and 72 h from reaching the target temperature range of 33 ± 1 °C. All patients diagnosed with an AMI at the immediate coronary angiogram (CAG)—18 in the 24-h group and 25 in the 48-h group—underwent PCI with stent implantation. There were no stent thromboses.

**Results:**

Both the hs-cTnT and hs-cTnI changes over time were highly influenced by the cause of OHCA (AMI vs. non-AMI). In contrast to non-AMI patients, both troponins remained elevated at 72 h in AMI patients. There was no difference between the two time-differentiated TTM groups in the kinetics for the two troponins.

**Conclusion:**

In comatose OHCA survivors with an aetiology of AMI levels of both hs-cTnI and hs-cTnT remained elevated for 72 h, which is in contrast to the well-described kinetic profile of troponins in normotherm AMI patients. There was no difference in kinetic profile between the two high sensitive assays. Different duration of TTM did not influence the kinetics of the troponins.

*Trial registration*: Clinicaltrials.gov Identifier: NCT01689077, 20/09/2012.

**Supplementary Information:**

The online version contains supplementary material available at 10.1186/s12872-022-02778-4.

## Introduction

Rise and fall of troponin levels following a period of ischemia provide important information about diagnosis of acute myocardial infarction (AMI) and infarct size [1, 2]. Elevated troponins are also observed in patients with out-of-hospital cardiac arrest (OHCA) without myocardial infarction. However, this elevation is modest and normalizes early [3] and the kinetic profile in this subset of patients with OHCA is supposed to be distinct from that after AMI [4].

Although mild hypothermia, now referred to as targeted temperature management (TTM) at 33–36 °C, for 24 h in comatose survivors after OHCA has become standard of care to limit the anoxic brain injury [5, 6], the kinetics of high-sensitive troponins and the effect of TTM on a potentially underlying AMI in this setting has not been extensively evaluated [7]. Experimental studies have shown that TTM induced before reperfusion of the acute coronary occlusion may reduce myocardial infarct size [8, 9]. A pilot study also indicated that patients with large ST segment elevation myocardial infarction (STEMI) benefit from short-term hypothermia induced before PCI with a subsequent reduction of infarct size [10]. Cautious interpretation of sub-group analyses in a recent study may indicate a favourable outcome with the use of TTM [11]. Moreover, in the Chill AMI trial there was a lower incidence of heart failure and a possible effect in patients with early anterior ST-segment elevation myocardial infarctions [12].

The aim of the present study was to explore and compare the kinetic pattern of two high-sensitivity troponins; hs-cTnT and hs-cTnI, in OHCA patients, with and without AMI, undergoing TTM at 33 ± 1 °C of two different durations in the intensive care unit (ICU).

## Methods

This study was a pre-specified explorative sub-study of the”Targeted Temperature Management for 48 vs 24 Hours and Neurological Outcome After Out-of-Hospital Cardiac Arrest: A Randomized Clinical Trial” (TTH48) in comatose OHCA survivors enrolled at 2 specific centres from February 2013 to June 2016 (ClinicalTrials.gov Identifier: NCT01689077) [13]. In brief, the TTH48 trial was an investigator-initiated, blinded-outcome-assessor, parallel, multicenter, randomized clinical trial, which evaluated effects of prolonged TTM for 48 h compared to standard 24-h TTM at 33 ± 1 °C. Inclusion criteria were OHCA with a presumed cardiac origin, sustained return of spontaneous circulation (ROSC), Glasgow Coma Score less than 8 and age older than 17 years and below 80 years. The full protocol has been described previously [14].

### Acute myocardial infarction

AMI was defined as thrombotic lesion identified at the immediate coronary angiography (CAG) necessitating a percutaneous coronary intervention (PCI).

### TTM

**Fig. 1:**
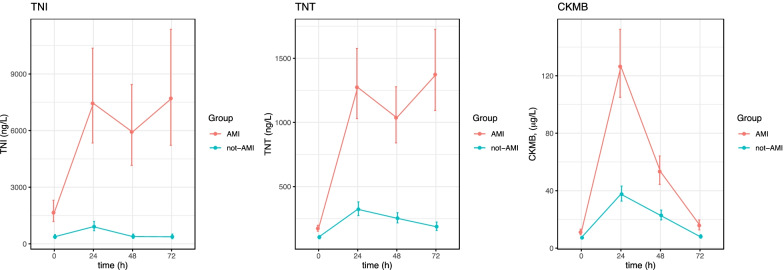
Kinetics of TnI, TnT and CK-mb in comatose survivors after OHCA. AMI compared to non-AMI. Pooled analyses of both treatment arms 24 and 48 h of TTM. Patients stratified by acute myocardial infarction (AMI) or non-AMI. hs-cTnT, hs-cTnI, CK-MB is log transformed, (mean ± SE). The units are ng/l. TNT; high sensitive troponin T, TNI; high sensitive troponin I, CK-MB; Creatine kinase MB, OHCA; out of hospital cardiac arrest, TTM; target temperature management

Patients were randomized to TTM maintained for either 24 or 48 h at 33 ± 1 °C (Fig. 1). After screening for eligibility, patients were randomly assigned in a 1:1 ratio to 1 of the 2 study groups, using a web-based central randomization procedure provided by the Department of Clinical Medicine, Aarhus University, Denmark. Both surface and invasive cooling methods with feedback were allowed. Cooling was initiated at admission to the ICU or at the cardiac catheterization laboratory. The patients were cooled as fast as possible to a target temperature of 33 ± 1 °C, and rewarming was conducted at a maximum rate of 0.5** °**C/h until a core temperature of 37˚C was reached.

### Clinical data collection

We collected study population characteristics such as gender, age, body mass index, previous medical history along with pre-hospital data following the Utstein template. Furthermore, we collected in-hospital data from the records in the cardiac catheterization laboratories and from the ICUs.

### Blood sampling and analyses

We collected blood samples for analyses at arrival, after 24 h, 48 h and 72 h. The Elecsys 2010 hs-Troponin T immunoassay (Roche Diagnostics, Penzberg, Germany) was used for measuring the hs-cTnT concentrations (cut-off point for the diagnosis of AMI: 14 ng/L [99th percentile of the upper reference limit (URL)]), while Troponin I was analysed using a commercially available high-sensitive cTnI STAT assay from Abbott Diagnostics, Architect i2000SR (Abbott Diagnostics, Illinois, USA) (immunochemistry) with a lower limit of detection of 1.6 ng/l assay for measuring hs-cTnI concentration (cut-off point: 15 ng/L for women and 30 for men [99th percentile of the upper reference limit (URL)]). Additionally, the measurements of CK-MB were carried out as a supplemental option for detecting myocardial injury and diagnosing myocardial infarction. The ARCHITECT STAT CK-MB immunoassay (Abbott Laboratories, Lake Bluff, Ill.) was applied for the measurement of the CK-MB concentrations. (cut-off points: 4.0 mg/L URL for women and 7.0 mg/L URL for men). We also measured and analysed serum creatinine at admission, after 24 h, 48 h and 72 h.

### Statistics

Data were managed using Research Electronic Data Capture; REDCap [15, 16]. Baseline characteristics were compared between treatment groups with Wilcoxon rank-sum test, or chi square test.

The influence of time (“pharmacokinetics”) and treatment group assignment (TTM duration) on hs-cTnT and hs-cTnI levels were addressed with linear mixed effects model analyses also taking into account the cause of cardiac arrest (AMI or non-AMI) as well as the serum creatinine level, because it is believed that renal filtration is responsible for clearance of TnT and TnI. A linear mixed effects model was applied to answer whether serum creatinine level was affected by treatment group assignment and how the serum-concentration changed over time. All statistical analyses were done using R (Version 3.5.1 using Rstudio version 1.1.453) with the *tableone*, *nlme* and *lme4* packages installed. *p* < 0.05 was considered statistically significant.

## Results

One hundred and fourteen patients were included in 2 cardiac arrest centers (Aarhus DK and Stavanger NO) between February 2013 and June 2016, Patients diagnosed with AMI (n = 43) had an angiographically documented thrombotic lesion, and all of these patients underwent subsequent PCI (18 in the 24-h group and 25 in the 48-h group). At baseline, there were no differences in biochemistry or demographics of AMI versus non-AMI patients except for S-creatinine levels, which was statistically significantly lower in the patients with AMI as aetiology compared to patients with no AMI. Additionally there was a borderline difference in prevalence of diabetes (Table 1). There were no stent thromboses.

**Table 1:** Baseline demographics and admission lab values in patients with Non-AMI and AMI

	Stratified by group		
	Non-AMI	AMI	*p*
n	71	43	
Age (years)	61 [53, 68]	65 [55, 70]	0.166
Male gender (%)	60 (84.5)	38 (88.4)	0.766
BMI (kg/m^2^)	27 [24, 29]	28 [25, 29]	0.382
Diabetes mellitus (%)	18 (25.4)	4 (9.3)	0.063
Smoking (%)			0.862
Never	13 (22.0)	8 (20.5)	
Present	21 (35.6)	16 (41.0)	
Prevoius	25 (42.4)	15 (38.5)	
Hypercholesterolemia (%)			0.877
Missing	1 (1.4)	1 (2.3)	
No	46 (64.8)	29 (67.4)	
Yes	24 (33.8)	13 (30.2)	
Hypertension (%)	35 (49.3)	20 (46.5)	0.924
Previous AMI (%)	16 (22.5)	9 (20.9)	1
Alcohol abuse (%)			0.077
Missing	5 (7.0)	3 (7.0)	
No	64 (90.1)	34 (79.1)	
Yes	2 (2.8)	6 (14.0)	
CPR performed (%)	61 (85.9)	39 (90.7)	0.646
Primary rhythm (%)			0.516
Missing	7 (9.9)	3 (7.0)	
Non-shockable	9 (12.7)	3 (7.0)	
Shockable	55 (77.5)	37 (86.0)	
No flow time (minutes)	1 [0, 1]	0 [0, 1]	0.49
Low flow time (minutes)	17 [12, 28]	21 [15, 27]	0.361
ROSC (minutes)	20 [13, 28]	22 [16, 27]	0.438
LUCAS (%)			0.187
Missing	0 (0.0)	1 (2.3)	
No	46 (64.8)	22 (51.2)	
Yes	25 (35.2)	20 (46.5)	
ROSC to CAG (minutes)	79 [58, 109]	79 [56, 104]	0.988
48 h TTM treatment (%)	36 (50.7)	25 ( 58.1)	0.563
TNI (ng/l)	237 [86, 1887]	1661 [180, 6407]	0.004
TNT (ng/l)	105 [48, 213]	163 [96, 351]	0.012
CKMB (ug/l)	7 [4, 13]	8 [5, 22]	0.085
Lactate (mmol/l)	3 [2, 7]	3 [2, 5]	0.617
Creatinine (mmol/l)	105 [88, 125]	91 [82, 108]	0.012
SAPSII	52 [47, 59]	50 [44, 58]	0.537
Surface cooling (%)	20 (28.2)	14 (32.6)	0.775

*CPR* cardiopulmonary resuscitation, *ROSC* return of spontaneous circulation, *CAG* coronary angiography, *AMI* acute myocardial infarction. Continuous variables are reported as median [interquartile range]

**Fig. 2:**
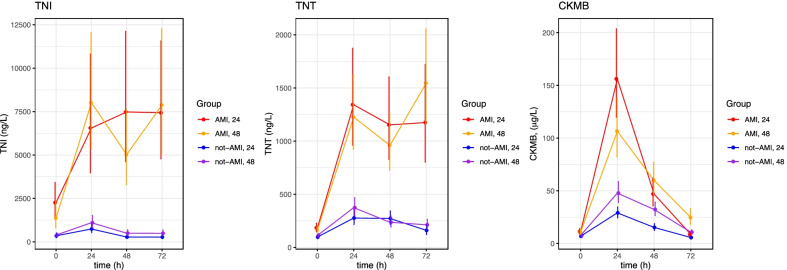
Kinetics of TnI, TnT and CK-mb in comatose survivors after OHCA treated with TTM in the TTH48 trial stratified by cause of event (AMI vs. non-AMI) and treatment group (TTM 24 h vs. TTM 48 h). Patients stratified by AMI and length of TTM, (mean ± SE). hs-cTnT, hs-cTnI, CK-MB. The units are ng/l. TNI; high sensitive troponin I, TNT; high sensitive troponin T, CK-MB; Creatin kinase MB, OHCA; out of hospital cardiac arrest, TTM; target temperature management

The presence of AMI highly altered the general level and kinetics of both troponins (Fig. 1). In this group specifically, there was no statistically significant difference in the kinetic pattern of the 2 troponins between the two time-differentiated TTM groups (Fig. 2). However, both hs-cTnT and hs-cTnI remained elevated at 72 h in both TTM groups with AMI. This is different from the well-known kinetic profile in normothermic patients with AMI in whom both troponins are almost halved at 50 h despite the double dome curve of TNT.

**Fig. 3:**
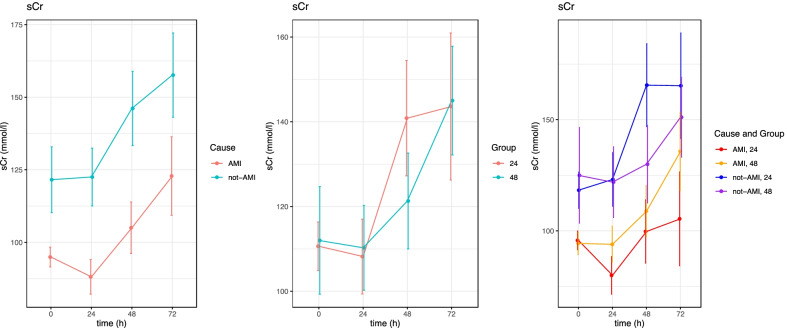
Changes in serum creatinine during 72 h as a function of aetiology (AMI or not AMI), treatment group (TTM 24 h vs. TTM 48 h) and the combination of these. Patients stratified by AMI or no-AMI, (mean ± SE). sCr; serum creatinine, AMI; acute myocardial infarction, TTM; target temperature management

Level of serum creatinine were, at each time point, statistically significantly *lower* in the patients with AMI as aetiology compared to patients with no AMI (Fig. 3). Level of serum creatinine highly altered the general level of both troponins. Although affecting the general troponin level, serum creatinine was not in itself affected by the treatment group.

**Fig. 4:**
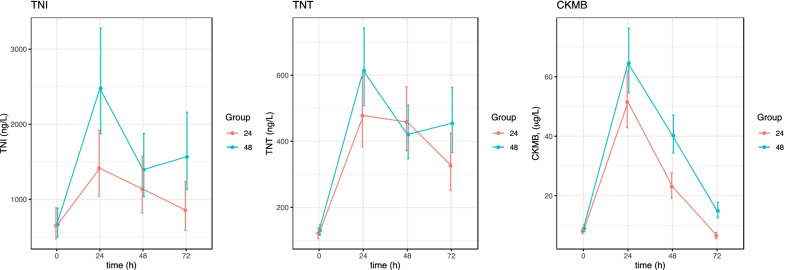
Kinetics of TnI, TnT and CK-mb in comatose survivors after OHCA treated with TTM in the TTH48 trial. Duration of TTM. Patients stratified by treatment group, (mean ± SE). The units are ng/l. TNI; high sensitive troponin I, TNT; high sensitive troponin T, MB; Creatine kinase MB, TTM; target temperature management

Different duration of TTM did not influence the kinetics of the troponins (Fig. 4).

**Fig. 5:**
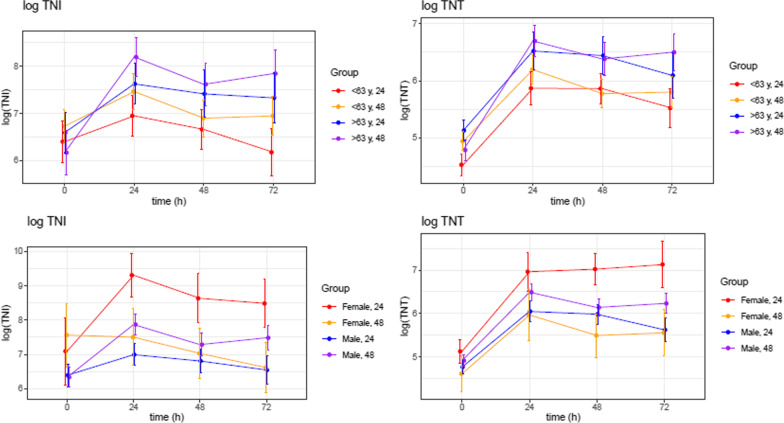
Effect of age > 63 and female gender on the levels troponins. Patients stratified by age and gender, (mean ± SE). The units are ng/l. TNI; high sensitive troponin I, TNT; high sensitive troponin T

**Fig. 6:**
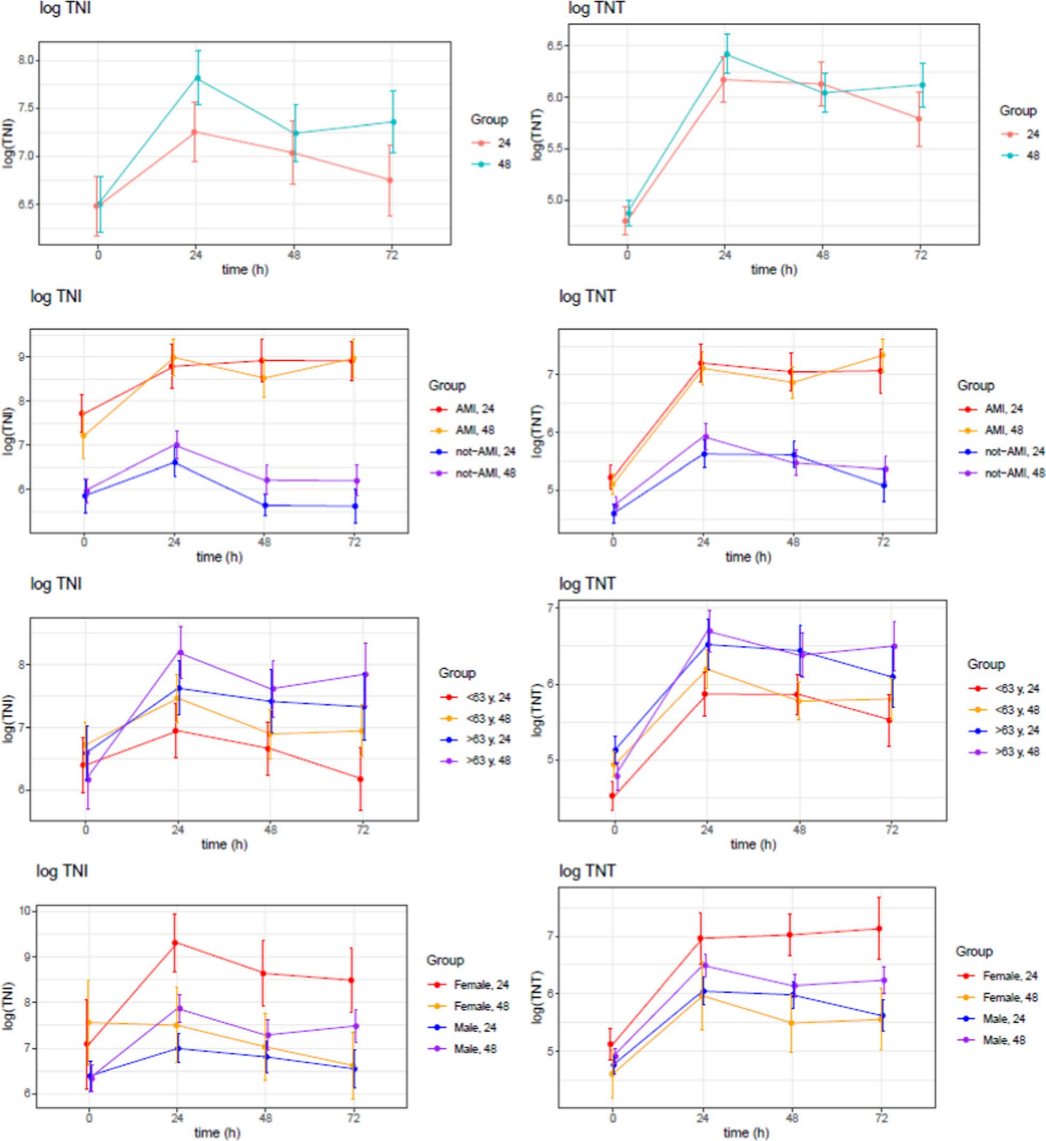
Summary of results for TNI, TNT and CK-MB, treatment length, AMI versus non ami, age and gender. The units are ng/l. TNI; high sensitive troponin I, TNT; high sensitive troponin T, AMI; acute myocardial infarction. (mean ± SE)

Female gender and age > 63 was associated with higher levels of troponins (Fig. 5). However, a higher number of patients with AMI as an aetiology mainly drove the effect of age for OHCA in the elderly. Figure 6 is high lightening the summary of the study.

## Discussion

The main finding in the current study was that both levels of hs-cTnT and hs-cTnI remained elevated at time point 72 h in OHCA patients diagnosed with AMI undergoing TTM. This contrasts the well-known kinetic profile of troponins in patients with AMI not undergoing TTM [17]. There was no difference in kinetics between the two troponins.

Further, we found that serum level of creatinine was a major predictor for alterations of the kinetics of both troponins. Finally, we found that the kinetic profiles of hs-cTnT and hs-cTnI were not affected by length of TTM (24 h and 48 h of TTM at 33 ± 1 °C).

### Comparison with previous studies

The typical double doom kinetic curve of hs-cTnT in normothermic patients following AMI is caused by the early release from a 'cytosolic pool' contributing to an initial peak with a second peak, which also reflects infarct size, caused by degradation of the contractile apparatus ('structural pool'). In contrast to this, hs-cTnI profile is monophasic, lacking a second, late peak. However, both troponins are falling after 24–48 h in STEMI patients treated with primary PCI.

In the current study levels of both troponins remained elevated at 72 h. These findings of persistent elevated levels of troponins during TTM in patients with AMI, indicating additional myocardial injury, are in contrast to a pooled analysis of six randomized trials which indicated a reduction in infarct size in anterior STEMI patients who were cooled to < 35 °C at the time of reperfusion, assessed by either single-photon emission computed tomography (SPECT) or cardiac magnetic resonance imaging (CMR) [18]. However, a recently published report concluded that out-of-hospital induced cooling, as an adjunct to primary percutaneous coronary intervention did not improve myocardial salvage as assessed with CMR in patients with STEMI [19]. Additionally, in the Cool-AMI study, a reduction of body core temperature to 33.3 °C before primary PCI in patients with anterior STEMI was associated with increased risk for adverse events [20]. Concomitant reduced fall in levels of CK-MB in the 48 h treatment arm compared with the 24-h arm in a sub-study of the TTH 48 trial further support the potential deleterious effects on the myocardium of TTM for comatose survivors of OHCA in patients with AMI [21].

### Renal function and troponin levels

In addition to the obvious typical rise and fall due to AMI, troponin values have also been shown to rise and fall with increasing and decreasing kidney function, suggesting renal clearance as a contributing factor of the rise and fall independence of myocardial injury [22]. In Chronic Renal Failure (CRF) patients, hs-cTnT increases over time as renal function decreases. Each 15 mL/min/1.73 m^2^ lower mean estimated glomerular filtration rate (GFR) has been shown to be associated with a 23% higher baseline hs-cTnT and 9% steeper increase in hs-cTnT in stable Chronic renal disease (CRD) stage 4–5 patients [23]. In the current study, level of serum creatinine per se also had a significant impact on troponin levels. However, admission serum creatinine was lower in the AMI group indicating that the the observed persistent elevated levels of troponins in this group are unlikely to be related to kidney function only.

### Hypothermia and renal function

Acute kidney injury (AKI) is common in OHCA, depending on definition and patient selection [24, 25]. The post cardiac arrest syndrome is characterised by multi-organ dysfunction as a result of an acute inflammatory response and persistent haemodynamic instability aggravated by ischaemia–reperfusion injury and on-going shock aggravating renal injury [26]. It is suggested that TTM impacts the development of AKI as indicated in a sub-study of the *Hypothermia after Cardiac Arrest Study*, in which hypothermia to 33 °C was associated with a reduced calculated glomerular filtration rate [27]. However, the evidences are diverging, and mild therapeutic hypothermia does not seem to be reno-protective [28]. Serum creatinine rose in both groups, but there were no differences between treatment groups indicating any additional drop in renal function in the 48-h group.

### Contrast induced nephropathy

It has been speculated that the sustained levels of troponins may be explained by an additional aggravation of renal failure due to early coronary angiography (CAG) and PCI in OHCA survivors with AMI. Yet, in a sub-study of the TTH48 trial on AKI there was no association between prolonged TTM at 33 °C and risk of AKI during the first seven days of admission [29]. Moreover, recent studies show that early CAG-PCI does not increase the risk of AKI per se. In the COACT trial, the incidence of AKI and need for renal-replacement therapy did not differ between early versus late CAG-PCI [30]. Furthermore, in the post hoc analysis of the targeted temperature management (TTM) trial, early CAG-PCI was even associated with less AKI [31].

There is thus no clear evidence of an acute negative effect of CAG on renal function in TTM explaining the sustained elevated levels of troponins in the subset of patients with AMI undergoing CAG and PCI. This finding is also supporting the hypothesis of increased myocardial injury as a source for sustained elevation of troponins also in patients with CRF. There is emerging evidence that increases in cTnT in patients without clinical symptoms of AMI with end stage renal disease indicates subclinical myocardial necrosis or injury[32, 33]. This is partly based on the findings of no difference between patients with normal renal function or end stage renal disease (ESRD) in the elimination half-life and apparent half-life of serum cTnI during myocardial necrosis [34]. The intact troponin molecule is large, and it is unlikely that the kidneys are primarily responsible for clearance from serum. However, work by Diris et al. suggests that the troponin molecule is degraded into smaller fragments, which can be detected by the assays and are small enough to be filtered by the kidneys. This mechanism may contribute to the elevation of troponin in severe renal failure [35].

The mechanisms for the sustained troponin plateau phase in the present AMI patients undergoing TTM may thus be plural. Reduced renal clearance is suggested to contribute to the sustained levels of troponins in some patients. However, we found no association between prolonged TTM at 33 °C and the risk of AKI. Therefore, increased myocardial necrosis cannot be excluded. This may be reinforced by too low target mean arterial pressure increasing troponin levels as shown in a study on 120 OHCA patients with AMI and shock [36]. Additionally, levels of Hs-TnT have been shown to be a marker of poor prognosis after OHCA [37].

Both cTnI and cTnT are released from necrotic myocardium as intact proteins and as degradation products [38]. Whether the substantial biochemical differences between cTnI and cTnT including molecular weight between cTnI (23 kDa) and cTnT (35 kDa) could result in different diagnostic and prognostic performances for both biomarkers are not well explored [39]. In the current study this difference in pharmacokinetics was not evident. This may be due to the myo-fibrillar injury and mitochondrial oedema and reversible ultrastructural findings in medically and experimentally induced moderate or deep hypothermic blood cardioplegia [40, 41].

Finally, sustained release of troponins might be caused by stent thrombosis. However, there were no clinical indication of acute stent thrombosis. This is also in accordance with a recent study showing that the incidence of stent thrombosis was low in OHCA survivors undergoing state of the art PCI [42]. On the other hand, although the confirmation of a diagnosis of stent thrombosis using troponin dynamics will be challenging, one would expect a new substantial peak in troponin levels and signs of acute ischemia in the ECG.

The findings of remained elevated troponins in comatose OHCA survivors undergoing TTM might indicate a potential risk for cooling induced myocardial damage which is associated with multi-organ failure. Recently the TTM2 trial showed that targeted hypothermia did not lead to a lower incidence of death by 6 months than targeted normothermia in patients with coma after out-of-hospital cardiac arrest [43]. The results were broadly consistent with the results of the previous TTM trial. The combined results of the two trials imply a low likelihood of any meaningful clinical improvement with hypothermia as compared with normothermia, since 36 °C may be considered to be the lower boundary of normothermia. In the TTM2 trial arrhythmias causing hemodynamic compromise were more common in the hypothermia group than in the normothermia group. Possible reasons for this include electrolyte disturbances, fluid status, and a temperature effect on cardiac myocytes [44].

### Strengths and limitations

This study had a prospective design with pre-set time-points for analyses of troponins. It was performed in two cardiac arrest centres with well-developed systems for all post cardiac arrest patients. On the other hand, there was a relative low number of patients included and a limited number of time points to obtain a more precise kinetic profile.

## Conclusions

In OHCA survivors we found no difference in the pharmacokinetics of two high-sensitive troponins during TTM at 33 °C for 24 or 48 h. Both troponins remained elevated at 72 h in the patients undergoing PCI for AMI unlike the kinetic profile for these troponins in normothermic patients with AMI. The usefulness of hs-troponin I and T might thus not be appropriate for diagnosing new coronary events in these patients.

## Supplementary Information

**Additional file 1**. Raw Data Set.

## Data Availability

All data are available in PDF format in “Additional file 1”.

## Abbreviations

AKI: Acute kidney injury
AMI: Acute myocardial infarction
CAG: Coronary angiography
CRF: Chronic Renal Failure
CK-MB: Creatine kinase MB
CMR: Cardia magnetic resonance imaging
CRD: Chronic renal disease
ESRD: End stage renal disease
GFR: Glomerular filtration rate
hs-cTnI: High-sensitivity troponin I
hs-cTnT: High-sensitivity troponin T
ICU: Intensive care unit
OHCA: Out-of-hospital cardiac arrest
PCI: Percutaneous coronary intervention
REDCap: Research Electronic Data Capture
ROSC: Return of spontaneous circulation
SPECT: Single-photon emission computed tomography
STEMI: ST segment elevation myocardial infarction
TTM: Targeted temperature management
URL: Upper reference limit
