# Supplementary material for: Kinetics of 2 different high-sensitive troponins during targeted temperature management in out-of-hospital cardiac arrest patients with acute myocardial infarction: a post hoc sub-study of a randomised clinical trial
Source: BMC Cardiovasc Disord. 2022 Jul 30;22:342. doi: 10.1186/s12872-022-02778-4 (PMC9339199; doi:10.1186/s12872-022-02778-4)
Supplement: Supplementary file 1 — Additional file 1. Raw Data Set. [file 12872_2022_2778_MOESM1_ESM.pdf]

Raw Data Set

Randomisation number	Time	Troponin I	Troponin T	Creatinine	Creatine kinase-MB	Group	Age	Sex	Body Mass Index	SAPSII
**17002**	0	439	219	118		48	48	Male	25.826447	55
**17002**	24	5961	517	78	239	48	48	Male	25.826447	55
**17002**	48	1833	377	75	200	48	48	Male	25.826447	55
**17002**	72		302	80	101	48	48	Male	25.826447	55
**17003**	0	16	32	122	1	24	51	Male	26.874496	64
**17003**	24	373	214	83	16	24	51	Male	26.874496	64
**17003**	48	144	195	104	3	24	51	Male	26.874496	64
**17003**	72		59	108		24	51	Male	26.874496	64
**17004**	0	47	51	72		48	57	Male	23.4375	52
**17004**	24	275	139	36	33	48	57	Male	23.4375	52
**17004**	48	103	76	35	24	48	57	Male	23.4375	52
**17004**	72	90	46	48	7	48	57	Male	23.4375	52
**17005**	0	545	329	105	14	24	43	Male	24.691359	53
**17005**	24	4916	795	90	76	24	43	Male	24.691359	53
**17005**	48	1180	324	104	32	24	43	Male	24.691359	53
**17005**	72	441	249	94	8	24	43	Male	24.691359	53
**17006**	0	44106		60	184	48	57	Female	24.489796	67
**17006**	24	71090	3704	53	170	48	57	Female	24.489796	67
**17006**	48	22072	1912	55	63	48	57	Female	24.489796	67
**17006**	72		2369	64	13	48	57	Female	24.489796	67
**17007**	24	458	845	80	35	48	25	Female	30.864197	56
**17007**	48	252	204	81	10	48	25	Female	30.864197	56
**17007**	72	105	207	85	3	48	25	Female	30.864197	56
**17101**	0	81	77	128	2	48	59	Female	30.040817	53
**17101**	24	512	159	55	16	48	59	Female	30.040817	53
**17101**	48	266	111	58	8	48	59	Female	30.040817	53
**17101**	72	369	149	73	14	48	59	Female	30.040817	53
**17203**	0	50		104	3	48	77	Male	35.493828	64
**17203**	24	1098	345	73	40	48	77	Male	35.493828	64
**17203**	48	442	192	69	31	48	77	Male	35.493828	64
**17203**	72	637	259	74	3	48	77	Male	35.493828	64
**17204**	48	4170	189	90	9	48	63	Female	26.296566	56
**17204**	72		130	93	3	48	63	Female	26.296566	56
**17205**	0	1102	551	70	21	24	61	Female	34.602077	60
**17205**	24	88904	6819	89	600	24	61	Female	34.602077	60
**17205**	48	45914	4621	77	85	24	61	Female	34.602077	60
**17205**	72		4649	71	52	24	61	Female	34.602077	60
**72001**	0	667	120	109	2,9	24	48	Male	29.218407	41
**72001**	24	6401	834	78	49,2	24	48	Male	29.218407	41
**72001**	48	2895	458	73	17,9	24	48	Male	29.218407	41
**72001**	72	1107	445	77	3,8	24	48	Male	29.218407	41
**72003**	0		195	119	3,8	24	50	Male	24.930748	39
**72003**	24	748	191	110	17,3	24	50	Male	24.930748	39
**72003**	48	221	155	135	6	24	50	Male	24.930748	39
**72003**	72	90	72	120	2,2	24	50	Male	24.930748	39
**72004**	0	1936	160	84	6,1	48	43	Female	26.446281	42
**72004**	24	687	202	52	53,3	48	43	Female	26.446281	42
**72004**	48	325	148	48	44,2	48	43	Female	26.446281	42
**72004**	72	1796	284	63	26,2	48	43	Female	26.446281	42

**72006**	0	1051	151	99	5,8	48	54	Male	21.604939	40
**72006**	24		303	81	28,5	48	54	Male	21.604939	40
**72006**	48	343	185	90	20,7	48	54	Male	21.604939	40
**72006**	72		249	118	7,4	48	54	Male	21.604939	40
**72007**	0		27	79	3,8	24	51	Male	24.251278	44
**72007**	24	34	29	92	8,2	24	51	Male	24.251278	44
**72007**	48		26	162	8,0	24	51	Male	24.251278	44
**72007**	72	4	10	113	5.1999998	24	51	Male	24.251278	44
**72009**	0	888.03497	94	102	3.7	48	46	Male	23.319452	39
**72009**	24	454.698	205	62	30.299999	48	46	Male	23.319452	39
**72009**	48	308.80499	147		23.5	48	46	Male	23.319452	39
**72009**	72	176.66901	96	85	6.6999998	48	46	Male	23.319452	39
**72010**	0	228.205	27	82	4.4000001	24	56	Male	29.218407	39
**72010**	24	60.681999	41	48	13.8	24	56	Male	29.218407	39
**72010**	48		48		5.3000002	24	56	Male	29.218407	39
**72010**	72	43.158001	32	63	3.2	24	56	Male	29.218407	39
**72011**	0	1654.1331	830	62	65.400002	48	59	Male	24.835646	58
**72011**	24		2270	78	303.39999	48	59	Male	24.835646	58
**72011**	48		1285	82		48	59	Male	24.835646	58
**72012**	0	27241.834	150	91		24	57	Male	28.066423	48
**72012**	24		2981		288	24	57	Male	28.066423	48
**72012**	48	11318.291	1758		58.5	24	57	Male	28.066423	48
**72012**	72	4385.0381	1476	75	8.6000004	24	57	Male	28.066423	48
**72013**	0	4278.2192		84		48	23	Male	22.530613	35
**72013**	24	440.43799	201	55	20.6	48	23	Male	22.530613	35
**72013**	48	103.069	85		16	48	23	Male	22.530613	35
**72013**	72	66.862	68	76	4.5999999	48	23	Male	22.530613	35
**72015**	0	948.909	24			48	42	Female	23.529411	40
**72015**	24	1623.835	67	39	8.6999998	48	42	Female	23.529411	40
**72015**	48	1234.202	47		6.5999999	48	42	Female	23.529411	40
**72015**	72	1138.741	43	62	2.5	48	42	Female	23.529411	40
**72016**	0	184.63499	130	150	11.1	24	51	Male	24.930748	62
**72016**	24	20829.922	2695	197	242.5	24	51	Male	24.930748	62
**72016**	48		1672		136.89999	24	51	Male	24.930748	62
**72016**	72		1142	219	37.599998	24	51	Male	24.930748	62
**72017**	0	1066.994	558	172	5.5	48	44	Male	29.320988	48
**72017**	24	487.664	201	106	27.9	48	44	Male	29.320988	48
**72017**	48	186.63	105		29.4	48	44	Male	29.320988	48
**72017**	72	215.838	79	109	11.7	48	44	Male	29.320988	48
**72018**	0	196.349	34	54	2.3	48	53	Female	22.857143	42
**72018**	24	48.294998	36	44	7	48	53	Female	22.857143	42
**72018**	48	24.841	30		6.1999998	48	53	Female	22.857143	42
**72018**	72	31.330999	26	71	2.8	48	53	Female	22.857143	42
**72019**	0		175		9.3000002	24	47	Male	26.31579	43
**72019**	24	2767.2891	671	69	103.5	24	47	Male	26.31579	43
**72019**	48	3228.437	558		21.6	24	47	Male	26.31579	43
**72019**	72		485	75	3.9000001	24	47	Male	26.31579	43
**72020**	0	236.381	114	88	6.5	24	58	Male	26.566839	39
**72020**	24	527.72803	149	45	21.200001	24	58	Male	26.566839	39
**72020**	48		1140		125.6	24	58	Male	26.566839	39
**72020**	72	33655.324	2510	91	30.700001	24	58	Male	26.566839	39

**72021**	0	120.214	21	92	2.5999999	24	58	Male	23.671253	36
**72021**	24	1065.465	127	155	24.299999	24	58	Male	23.671253	36
**72021**	48	2014.99	161		13.2	24	58	Male	23.671253	36
**72021**	72		110	142	3.2	24	58	Male	23.671253	36
**72023**	0	72.447998	71	80	3.2	24	54	Female	35.918365	69
**72023**	24		271		25.799999	24	54	Female	35.918365	69
**72023**	48	605.68597	318		15.6	24	54	Female	35.918365	69
**72023**	72			162		24	54	Female	35.918365	69
**72026**	0	182.593	132	58	6	48	53	Male	20.549887	48
**72026**	24		1787	40	600	48	53	Male	20.549887	48
**72026**	48	49794.629	3142			48	53	Male	20.549887	48
**72026**	72	71076.016	3533	55	165.3	48	53	Male	20.549887	48
**72027**	0	3211.2859	35	107	5	24	54	Male	26.566839	
**72027**	24	2047.942	452	80	73.599998	24	54	Male	26.566839	
**72027**	48		407		35.299999	24	54	Male	26.566839	
**72027**	72	1311.762	252	71	4.5	24	54	Male	26.566839	
**72028**	0	483.75601	127	90	18.6	48	56	Male	22.159109	51
**72028**	24	13097.068	3117		273.5	48	56	Male	22.159109	51
**72028**	48		1628		158.89999	48	56	Male	22.159109	51
**72028**	72		1556	409	41.200001	48	56	Male	22.159109	51
**72029**	0	9924.2217	58	105		24	34	Male		32
**72029**	24	4628.9741	1269	137	29.799999	24	34	Male		32
**72029**	48		452		9.6999998	24	34	Male		32
**72029**	72	433.81799	322	108	1.6	24	34	Male		32
**72030**	0		56	77	7.9000001	24	43	Male	19.284149	42
**72030**	24	7872.0381	838	47	416.89999	24	43	Male	19.284149	42
**72030**	48		632		96.699997	24	43	Male	19.284149	42
**72030**	72	2077.6899	279	54	16.9	24	43	Male	19.284149	42
**72031**	0	357.806	241	113	5.8000002	48	53	Male	30.18959	42
**72031**	24	262.591	171	63	20.700001	48	53	Male	30.18959	42
**72031**	48		99		14.4	48	53	Male	30.18959	42
**72031**	72	81.630997	96	77	3.5	48	53	Male	30.18959	42
**72033**	0	68343.828	222		19.200001	24	37	Male	23.84803	51
**72033**	24	38081.477	4032	210	329	24	37	Male	23.84803	51
**72033**	48		2273		143.10001	24	37	Male	23.84803	51
**72033**	72	2269.884	651	231	39.299999	24	37	Male	23.84803	51
**72034**	0	42260.016	780	106	123.8	48	43	Male	29.320988	47
**72034**	24	53596.047	7699	119		48	43	Male	29.320988	47
**72034**	48	41558.641	5039		352.70001	48	43	Male	29.320988	47
**72034**	72	58737.363	8348	109	72.900002	48	43	Male	29.320988	47
**72035**	0	6333.6431	190	115	16.6	24	56	Male	29.320988	67
**72035**	24		1448	137	449.79999	24	56	Male	29.320988	67
**72035**	48	50000	3018		303.89999	24	56	Male	29.320988	67
**72035**	72	93114.898	3892	169	55.200001	24	56	Male	29.320988	67
**72036**	0	162.444	98	138	5.8000002	48	59	Male	33.240837	61
**72036**	24	272.772	100	163	15.1	48	59	Male	33.240837	61
**72036**	48	204.472	81		12.6	48	59	Male	33.240837	61
**72036**	72	306.91199	129	229	2.4000001	48	59	Male	33.240837	61
**72037**	0	172.31	136	108	6.6999998	24	48	Male	28.08901	40
**72037**	24	9551.1709	1184	73	182	24	48	Male	28.08901	40
**72037**	48	6005.5439	988		28	24	48	Male	28.08901	40

**72037**	72	2801.2549	864	82	5.0999999	24	48	Male	28.08901	40
**72038**	0	169.916	92	83	6.4000001	48	53	Male	23.62949	39
**72038**	24	2366.239	419	86	100.6	48	53	Male	23.62949	39
**72038**	48	3325.48	550		81.099998	48	53	Male	23.62949	39
**72038**	72	12278.786	773	245	137.89999	48	53	Male	23.62949	39
**72039**	0	467.29501	14	105		24	53	Male	27.440599	39
**72039**	24	94.063004	50	80	7.5999999	24	53	Male	27.440599	39
**72039**	48	19.833	43		3.8	24	53	Male	27.440599	39
**72039**	72	57.301998	17	92	2.2	24	53	Male	27.440599	39
**72040**	0	118.605	157	142	7.9000001	48	40	Male	24.376732	50
**72040**	24	45235.633	1943	189	131.10001	48	40	Male	24.376732	50
**72040**	48	29074.711	1005		97.699997	48	40	Male	24.376732	50
**72040**	72	14910.893	650	178	16.9	48	40	Male	24.376732	50
**72041**	0	7982.8882	425	94	18.4	24	49	Male	22.09317	51
**72041**	24	2172.7419	1056	149	44.799999	24	49	Male	22.09317	51
**72041**	48	3191.1841	1665		18.799999	24	49	Male	22.09317	51
**72041**	72	1128.665	1119	276	13.1	24	49	Male	22.09317	51
**72042**	0	48477.984	278	81	11.4	48	40	Female	29.411764	42
**72042**	24	21615.773	2543	77	289.20001	48	40	Female	29.411764	42
**72042**	48	16682.475	2120	81	155.3	48	40	Female	29.411764	42
**72042**	72	22000.807	2706	80	21.1	48	40	Female	29.411764	42
**72043**	0		59	125	4.9000001	48	55	Male	26.234568	39
**72043**	24	1636.28	644	119	60.400002	48	55	Male	26.234568	39
**72043**	48	608.59601	428		47.299999	48	55	Male	26.234568	39
**72043**	72		441	166	15	48	55	Male	26.234568	39
**72044**	0	23051.682	237	122	36.700001	48	52	Male	26.775511	54
**72044**	24	47498.355	4495	109		48	52	Male	26.775511	54
**72044**	48	23664.393	3792			48	52	Male	26.775511	54
**72044**	72	34838.73	3938	121	69.300003	48	52	Male	26.775511	54
**72045**	0	177.728	90	82	4.6999998	24	51	Male	29.916897	42
**72045**	24	486.61301	143	56	19.299999	24	51	Male	29.916897	42
**72045**	48	541.255	159		5.6999998	24	51	Male	29.916897	42
**72045**	72	274.556		69		24	51	Male	29.916897	42
**72101**	0	3195	106	88	7	48	59	Male	24.489796	52
**72101**	24	2074	495	94	43,5	48	59	Male	24.489796	52
**72101**	48	544	235	87	24,9	48	59	Male	24.489796	52
**72101**	72	645	298	160	5,8	48	59	Male	24.489796	52
**72102**	0	224.30299	195	336	9.8000002	24	53	Male	25.661152	68
**72102**	24	152.21201	193	352	13.4	24	53	Male	25.661152	68
**72102**	48	261.422	225		5.4000001	24	53	Male	25.661152	68
**72102**	72	289.69299	125	199	5.1999998	24	53	Male	25.661152	68
**72106**	0	1923.095	97	132	5.6999998	48	58	Male	30.864197	64
**72106**	24	1116.5129	589		45.599998	48	58	Male	30.864197	64
**72106**	48	567.31201	270		20.9	48	58	Male	30.864197	64
**72106**	72	718.90601	171	195	5.0999999	48	58	Male	30.864197	64
**72204**	0	4508	2381	102	322,2	48	65	Male	37.026398	42
**72204**	24	56626	5978	93	482,9	48	65	Male	37.026398	42
**72204**	48	40698	3516	96	230,5	48	65	Male	37.026398	42
**72204**	72	60880	8141	161	28,9	48	65	Male	37.026398	42
**72205**	0	237	365	113	2,5	24	70	Male	33.139755	50
**72205**	24	210	185	61	8	24	70	Male	33.139755	50

**72205**	48	121	205	65	2,4	24	70	Male	33.139755	50
**72205**	72	97	151	64	1,1	24	70	Male	33.139755	50
**72206**	0	2132	145	102	7,3	48	65	Male	24.835646	44
**72206**	24	2269	426	68	59,9	48	65	Male	24.835646	44
**72206**	48	660	184	62	30,7	48	65	Male	24.835646	44
**72206**	72	1061	258	70	7,1	48	65	Male	24.835646	44
**72207**	0	61442	124	78	6,9	24	69	Female	21.258503	46
**72207**	24	30191	2358	54	217,3	24	69	Female	21.258503	46
**72207**	48	30669	1815	76	46,5	24	69	Female	21.258503	46
**72207**	72	27383	3202	160	6,1	24	69	Female	21.258503	46
**72208**	0	6481	429	104	10,3	24	60	Male	24.691359	44
**72208**	24		637	67	41,8	24	60	Male	24.691359	44
**72208**	48	312	464	79	7,4	24	60	Male	24.691359	44
**72208**	72		332	76	1,9	24	60	Male	24.691359	44
**72209**	0	1661	195	108	12,6	48	71	Male	27.681662	56
**72209**	24	29601	2597	137	474,9	48	71	Male	27.681662	56
**72209**	48	19580	1724	140	363,9	48	71	Male	27.681662	56
**72209**	72	36418	2975	186	168,1	48	71	Male	27.681662	56
**72210**	0	604	125	84	6,3	48	68	Male	23.407509	42
**72210**	24	1273	332	53	45,3	48	68	Male	23.407509	42
**72210**	48	357	207	56	39,7	48	68	Male	23.407509	42
**72210**	72	337	185	75	13,5	48	68	Male	23.407509	42
**72211**	0	210	125	133	9,1	24	66	Male	28.66613	62
**72211**	24	11198	7512	288	134,6	24	66	Male	28.66613	62
**72211**	48		6129	436	75	24	66	Male	28.66613	62
**72211**	72	2901	3027	584	21,8	24	66	Male	28.66613	62
**72212**	0	134337		108	15,3	48	73	Male	27.777779	51
**72212**	24	41342	5449	168	473,8	48	73	Male	27.777779	51
**72212**	48	18639	3032	180	268,3	48	73	Male	27.777779	51
**72212**	72	30402	4061	261	49,8	48	73	Male	27.777779	51
**72213**	0		41	89	4,9	24	73	Male	27.777779	48
**72213**	24	27	39	51	17,1	24	73	Male	27.777779	48
**72213**	48	76	40	80	10,5	24	73	Male	27.777779	48
**72213**	72		26	73	1,7	24	73	Male	27.777779	48
**72214**	0	3733	414	54	4,4	48	63	Male	22.857143	60
**72214**	24	487	201	57	21,2	48	63	Male	22.857143	60
**72214**	48	264	147	72	14,1	48	63	Male	22.857143	60
**72214**	72	1226		108	14,8	48	63	Male	22.857143	60
**72215**	0	19	33	109	4,5	24	69	Male	23.836735	24
**72215**	24	107	98	77	16,3	24	69	Male	23.836735	24
**72215**	48	97	98	75	8,1	24	69	Male	23.836735	24
**72215**	72	36	40	79	3,5	24	69	Male	23.836735	24
**72216**	0	8526	337	86	48,4	48	72	Male	27.681662	75
**72216**	24	45991	3162	97	205,7	48	72	Male	27.681662	75
**72216**	48	27414	1571	94	91,2	48	72	Male	27.681662	75
**72216**	72	27308	2338	106	11,9	48	72	Male	27.681662	75
**72217**	0		38	142	2,6	24	62	Male	29.154518	53
**72217**	24	59	35	72	5,2	24	62	Male	29.154518	53
**72217**	48	62	39	94	2,2	24	62	Male	29.154518	53
**72217**	72	36	38	93	1,4	24	62	Male	29.154518	53
**72218**	0	2047	656	144	29,6	48	64	Male	27.744749	50

**72218**	24	40730	3095	150	234,8	48	64	Male	27.744749	50
**72218**	48	33302	3100	165	156,9	48	64	Male	27.744749	50
**72218**	72	31356	3630	211	16,3	48	64	Male	27.744749	50
**72219**	0	989	47	131	2,8	24	69	Male	29.407787	58
**72219**	24	5303	672	107	89,5	24	69	Male	29.407787	58
**72219**	48	5068	661	121	24,1	24	69	Male	29.407787	58
**72219**	72	2761	522	93	4,6	24	69	Male	29.407787	58
**72220**	0	45	88	90	12,7	48	72	Male	28.408163	51
**72220**	24	11305	2110	97	166,4	48	72	Male	28.408163	51
**72220**	48	5152	1554	106	112,4	48	72	Male	28.408163	51
**72220**	72	5013	1294	79	19,5	48	72	Male	28.408163	51
**72221**	0	1875	174	139	17,7	48	63	Male	29.065928	63
**72221**	24	27923	2740	236	250,6	48	63	Male	29.065928	63
**72221**	48		921	134	130,4	48	63	Male	29.065928	63
**72221**	72		508	119	42	48	63	Male	29.065928	63
**72222**	0	37	38	80	8	48	72	Male	24.930748	47
**72222**	24	150	78	60	21,8	48	72	Male	24.930748	47
**72222**	48	39	39	59	21,2	48	72	Male	24.930748	47
**72222**	72	34	29	63	9	48	72	Male	24.930748	47
**72223**	0		112	99	7,4	24	75	Female	32.270786	63
**72223**	24		466	164	35,8	24	75	Female	32.270786	63
**72223**	48		1537	275	97,5	24	75	Female	32.270786	63
**72223**	72	15701	1383	422	15,6	24	75	Female	32.270786	63
**72224**	0	71	99	107	5,6	24	69	Male	29.068796	48
**72224**	24	617	279	69	30,8	24	69	Male	29.068796	48
**72224**	48	274	187	83	16,9	24	69	Male	29.068796	48
**72224**	72	91	116	79	3,6	24	69	Male	29.068796	48
**72225**	0		426	91	46,3	24	67	Male	28.719723	71
**72225**	24	15286	2657	89	152,7	24	67	Male	28.719723	71
**72225**	48	10439	1668		45,3	24	67	Male	28.719723	71
**72225**	72	3197	913	74	8,7	24	67	Male	28.719723	71
**72226**	0		92	87	5,7	48	79	Male	34.285713	48
**72226**	24	86	69	49	37,2	48	79	Male	34.285713	48
**72226**	48	34	44	54	23,1	48	79	Male	34.285713	48
**72226**	72	35	39	65	7,2	48	79	Male	34.285713	48
**72227**	0	25	35	88	1,7	48	70	Male	28.405504	50
**72227**	24	33727	2681	75	600	48	70	Male	28.405504	50
**72227**	48	49764	4163	69	600	48	70	Male	28.405504	50
**72227**	72		4681	83	172,2	48	70	Male	28.405504	50
**72228**	0	2348	165	64	6,7	24	65	Female	23.4375	55
**72228**	24	3331	542	61	66,3	24	65	Female	23.4375	55
**72228**	48		502	70	15,9	24	65	Female	23.4375	55
**72229**	0	2266	196	102	6,2	24	71	Male	23.545706	47
**72229**	24		240	74	24,2	24	71	Male	23.545706	47
**72229**	48	396	155	76	12,9	24	71	Male	23.545706	47
**72229**	72	225	110	69	3,9	24	71	Male	23.545706	47
**72230**	0		106	99	3,5	48	69	Male	30.040817	50
**72230**	24	976	221	93	20	48	69	Male	30.040817	50
**72230**	48	474	147	95	13,9	48	69	Male	30.040817	50
**72230**	72	756	191	143	4	48	69	Male	30.040817	50
**72231**	0	25	38	162	6,7	24	61	Male	32.098766	53

**72231**	24	46	44	107	8,7	24	61	Male	32.098766	53
**72231**	48	104	65	143	6,9	24	61	Male	32.098766	53
**72231**	72	50	41	128	5,1	24	61	Male	32.098766	53
**72232**	0	4913	500	110	25,1	48	66	Male	29.387754	56
**72232**	24	13518	3012	246	122,4	48	66	Male	29.387754	56
**72232**	48		1843	314	77,4	48	66	Male	29.387754	56
**72232**	72	5813	1244	433	15,4	48	66	Male	29.387754	56
**72233**	0	88	593	63	17,4	24	69	Male	24.65591	72
**72233**	24	602	151	59	19,4	24	69	Male	24.65591	72
**72233**	48	142	120	81	10,3	24	69	Male	24.65591	72
**72233**	72		60	79	1,6	24	69	Male	24.65591	72
**72234**	0		84	99	9,8	48	65	Male	27.250887	51
**72234**	24	9932	1488	65	405,3	48	65	Male	27.250887	51
**72234**	48	3487	884	67	275,3	48	65	Male	27.250887	51
**72234**	72	4246	848	67	54,2	48	65	Male	27.250887	51
**72235**	0	38	33	124	2,6	48	73	Male	28.721121	53
**72235**	24	689	137	90	13,8	48	73	Male	28.721121	53
**72235**	48	493	88	94	11,5	48	73	Male	28.721121	53
**72235**	72	211	76	92	4,7	48	73	Male	28.721121	53
**72236**	0	76	29	101	1,6	48	61	Male	29.114527	57
**72236**	24	63	47	165	3,8	48	61	Male	29.114527	57
**72236**	48	38	38		3,4	48	61	Male	29.114527	57
**72236**	72	61	39	231	1,7	48	61	Male	29.114527	57
**72237**	0	3771	467	82	102,8	24	79	Male	23.510204	51
**72237**	24		1539	63	328,4	24	79	Male	23.510204	51
**72237**	48	12736	1705		112,7	24	79	Male	23.510204	51
**72237**	72	17207	2989	74	10	24	79	Male	23.510204	51
**72238**	0	5356	1620	108	137,5	24	68	Male	28.395061	61
**72238**	24		19554	188	600	24	68	Male	28.395061	61
**72238**	48	225259	18530	315	319,6	24	68	Male	28.395061	61
**72238**	72	179724	23750	421	31,6	24	68	Male	28.395061	61
**72239**	0	292		85	13,6	48	71	Male	24.913494	78
**72239**	24		3048	161	189,7	48	71	Male	24.913494	78
**72239**	48	4957	1869	217	127,5	48	71	Male	24.913494	78
**72239**	72	3418	1065	207	52,6	48	71	Male	24.913494	78
**72240**	0	234	222	103	33,9	48	63	Male	23.374725	53
**72240**	24		706	158	491,5	48	63	Male	23.374725	53
**72240**	48		463	204	357,4	48	63	Male	23.374725	53
**72240**	72	1744	408	250	76,6	48	63	Male	23.374725	53
**72241**	0		335	113	12,3	24	71	Male	25.142857	50
**72241**	24	1033	365	87	39,4	24	71	Male	25.142857	50
**72241**	48	1022	323	114	11	24	71	Male	25.142857	50
**72241**	72	658	330	104	3	24	71	Male	25.142857	50
**72242**	0	1565	356	62	21,6	48	70	Female	26.12245	47
**72242**	24	5898	846	58	77,3	48	70	Female	26.12245	47
**72242**	48	2840	784	128	42,2	48	70	Female	26.12245	47
**72242**	72	2331	615	142	6	48	70	Female	26.12245	47
**72243**	0		77	138	4,5	24	78	Male	31.020409	68
**72244**	0		1271	76	44,0	24	65	Female	27.777779	54
**72244**	24	12685	2770	226	167,3	24	65	Female	27.777779	54
**72244**	48	4749	2257	403	69,8	24	65	Female	27.777779	54

**72244**	72	1580	961	505	18,7	24	65	Female	27.777779	54
**72245**	0	688	426	121	24,9	24	64	Male	24.515596	64
**72245**	24	33518	2750	62	355,1	24	64	Male	24.515596	64
**72245**	48	32204	2939	75	99,6	24	64	Male	24.515596	64
**72245**	72	20706	3169	73	9,2	24	64	Male	24.515596	64
**72246**	0	3338	422	62	55,7	48	60	Male	24.489796	47
**72246**	24	25868	2211	67	309,5	48	60	Male	24.489796	47
**72246**	48	31144	2449	65	194,8	48	60	Male	24.489796	47
**72246**	72	18843	1968	70	102,2	48	60	Male	24.489796	47
**72247**	0	43	29	88	5	48	73	Male	32.432434	59
**72247**	24	229	101	71	30,3	48	73	Male	32.432434	59
**72247**	48	95	62	71	20	48	73	Male	32.432434	59
**72248**	0	5576	137	124	8,5	48	67	Male	33.60117	47
**72248**	24	11076	1779	84	152	48	67	Male	33.60117	47
**72248**	48	6416	1044	96	73,5	48	67	Male	33.60117	47
**72248**	72	9704	1272	120	12,2	48	67	Male	33.60117	47
**72249**	0	124	69	199	7	24	66	Male	23.374725	66
**72249**	24	277	105	178	29,7	24	66	Male	23.374725	66
**72249**	48	91	107	169	11,6	24	66	Male	23.374725	66
**72249**	72	110	99	172	2,3	24	66	Male	23.374725	66
**72250**	0		398	116	7,8	24	72	Male	29.320988	53
**72250**	24	466	366	162	19,4	24	72	Male	29.320988	53
**72250**	48	385	493	199	6,9	24	72	Male	29.320988	53
**72250**	72	169	295	231	2,3	24	72	Male	29.320988	53
**72251**	0	23	64	107	4,6	48	65	Male	30.555555	47
**72251**	24	434	251	154	24,7	48	65	Male	30.555555	47
**72251**	48	107	168	203	19,1	48	65	Male	30.555555	47
**72251**	72	80	105	202	9,9	48	65	Male	30.555555	47
**72252**	0	22570	117	121	21,7	24	66	Male		57
**72252**	24	9984	1385	85	165,1	24	66	Male		57
**72252**	48			92		24	66	Male		57
**72253**	0	1872	683	87	24,3	24	67	Male	35.188534	
**72253**	24	7283	1452	45	241,3	24	67	Male	35.188534	
**72253**	48	4885	945	60	64	24	67	Male	35.188534	
**72254**	0	115	211	82	7,8	48	72	Male	24.691359	52
**72254**	24	221	194	93	9,9	48	72	Male	24.691359	52
**72254**	48	127	215	109	4,1	48	72	Male	24.691359	52
**72254**	72	73	91	93	1,9	48	72	Male	24.691359	52
**72255**	0	1296	418	125	13,7	48	68	Male	28.731922	50
**72255**	24	1088	369	59	66,6	48	68	Male	28.731922	50
**72255**	48	2216	543	91	26,6	48	68	Male	28.731922	50
**72255**	72			71		48	68	Male	28.731922	50
**72256**	0		254	71	11	24	65	Male	28.727377	46
**72256**	24	44753	6857	64	566,5	24	65	Male	28.727377	46
**72256**	48	52518	6420	89	152,8	24	65	Male	28.727377	46
**72256**	72	18977	3230	76	11,4	24	65	Male	28.727377	46
**72257**	0	50	60	103	3,6	48	73	Male	28.727377	49
**72257**	24	2059	463	78	49,3	48	73	Male	28.727377	49
**72257**	48	1420	329	112	30,4	48	73	Male	28.727377	49
**72257**	72		311	132	6,4	48	73	Male	28.727377	49
**72258**	0	44	47	89	4,2	48	63	Male	30.59853	69

**72258**	24	905	312	130	19,2	48	63	Male	30.59853		69
**72258**	48	228	192	124	13,7	48	63	Male	30.59853		69
**72258**	72	270	158	153	4,4	48	63	Male	30.59853		69
**72302**	0	15	28	94	2,6	24	61	Male	27.777779		58
**72302**	24	102	78	168	9	24	61	Male	27.777779		58
**72302**	48	32	41	152	4,8	24	61	Male	27.777779		58
**72302**	72	11	26	149	2,5	24	61	Male	27.777779		58
**72303**	0	141	74	70	65,7	24	69	Male	29.320988		50
**72303**	24	309	76	52	93,4	24	69	Male	29.320988		50
**72303**	48	194	76	61	41,3	24	69	Male	29.320988		50
**72303**	72	192	46	56	7,8	24	69	Male	29.320988		50
**72304**	0		104	820	7,1	48	69	Male	26.287807		67
**72304**	24	269	257	534	18,7	48	69	Male	26.287807		67
**72304**	48	351	263	611	23,2	48	69	Male	26.287807		67
**72304**	72		330	389	8,3	48	69	Male	26.287807		67
**72306**	0	700	349	88	17,4	48	63	Male		32	57
**72306**	24		1015	158	62,6	48	63	Male		32	57
**72306**	48		805	221	46,4	48	63	Male		32	57
**72306**	72	2588	1051	331	15	48	63	Male		32	57
**72307**	0	219	247	96	8,1	24	78	Female	24.167162		59
**72307**	24	1422	491	122	49,4	24	78	Female	24.167162		59
**72307**	48	1374	493	170	34,7	24	78	Female	24.167162		59
**72307**	72	801	156	175	46,7	24	78	Female	24.167162		59
**72308**	0		31	168	4,9	48	70	Male	36.295921		56
**72308**	24	30215	1961	146	600	48	70	Male	36.295921		56
**72308**	48	42765	2925	169	600	48	70	Male	36.295921		56
**72308**	72	47752	4543	176	178,4	48	70	Male	36.295921		56
**72309**	0	49	55	96	6	48	60	Male	34.256054		57
**72309**	24	90	37	60	15,3	48	60	Male	34.256054		57
**72309**	48	137	52	68	15,4	48	60	Male	34.256054		57
**72309**	72	376	94	82	8,4	48	60	Male	34.256054		57
**72310**	0		323	121	35,6	48	67	Male	28.982006		56
**72310**	24	71985	10195	229	447,6	48	67	Male	28.982006		56
**72310**	48	43878	7834	316	264,4	48	67	Male	28.982006		56
**72310**	72	127267	9117	432	68	48	67	Male	28.982006		56
**72311**	0		169	108	16,8	24	62	Male	28.680111		72
**72311**	24	422	101	110	18,2	24	62	Male	28.680111		72
**72311**	48	175	57	109	6	24	62	Male	28.680111		72
